# PARS risk charts: A 10-year study of risk assessment for cardiovascular diseases in Eastern Mediterranean Region

**DOI:** 10.1371/journal.pone.0189389

**Published:** 2017-12-19

**Authors:** Nizal Sarrafzadegan, Razieh Hassannejad, Hamid Reza Marateb, Mohammad Talaei, Masoumeh Sadeghi, Hamid Reza Roohafza, Farzad Masoudkabir, Shahram OveisGharan, Marjan Mansourian, Mohammad Reza Mohebian, Miquel Angel Mañanas

**Affiliations:** 1 Isfahan Cardiovascular Research Center, Cardiovascular Research Institute, Isfahan University of Medical Sciences, Isfahan, Iran; 2 School of Population and Public Health, Faculty of Medicine, University of British Columbia, Vancouver, British Columbia, Canada; 3 Department of Epidemiology and Biostatistics, School of Public Health, Isfahan University of Medical Sciences, Isfahan, Iran; 4 Biomedical Engineering Department, Engineering Faculty, University of Isfahan, Isfahan, Iran; 5 Department of Automatic Control, Biomedical Engineering Research Center, Universitat Politècnica de Catalunya, BarcelonaTech (UPC), Barcelona, Spain; 6 Saw Swee Hock School of Public Health, National University of Singapore, Singapore, Singapore; 7 Department of Cardiology, School of Medicine, Tehran Heart Center, Tehran University of Medical Sciences, Tehran, Iran; 8 Cardiac Primary Prevention Research Center, Tehran Heart Center, Tehran University of Medical Sciences, Tehran, Iran; 9 Department of Neurology, School of Medicine, Tehran University of Medical Sciences, Tehran, Iran; 10 Rush Alzheimer’s disease Center; Rush University Medical Center, Chicago, Illinois, United States of America; 11 Biomedical Research Networking Center in Bioengineering, Biomaterials and Nanomedicine (CIBER-BBN), Barcelona, Spain; CUNY, UNITED STATES

## Abstract

This study was designed to develop a risk assessment chart for the clinical management and prevention of the risk of cardiovascular disease (CVD) in Iranian population, which is vital for developing national prevention programs. The Isfahan Cohort Study (ICS) is a population-based prospective study of 6504 Iranian adults ≥35 years old, followed-up for ten years, from 2001 to 2010. Behavioral and cardiometabolic risk factors were examined every five years, while biennial follow-ups for the occurrence of the events was performed by phone calls or by verbal autopsy. Among these participants, 5432 (2784 women, 51.3%) were CVD free at baseline examination and had at least one follow-up. Cox proportional hazard regression was used to predict the risk of ischemic CVD events, including sudden cardiac death due to unstable angina, myocardial infarction, and stroke. The model fit statistics such as area under the receiver-operating characteristic (AUROC), calibration chi-square and the overall bias were used to assess the model performance. We also tested the Framingham model for comparison. Seven hundred and five CVD events occurred during 49452.8 person-years of follow-up. The event probabilities were calculated and presented color-coded on each gender-specific PARS chart. The AUROC and Harrell’s C indices were 0.74 (95% CI, 0.72–0.76) and 0.73, respectively. In the calibration, the Nam-D’Agostino χ^2^ was 10.82 (p = 0.29). The overall bias of the proposed model was 95.60%. PARS model was also internally validated using cross-validation. The Android app and the Web-based risk assessment tool were also developed as to have an impact on public health. In comparison, the refitted and recalibrated Framingham models, estimated the CVD incidence with the overall bias of 149.60% and 128.23% for men, and 222.70% and 176.07% for women, respectively. In conclusion, the PARS risk assessment chart is a simple, accurate, and well-calibrated tool for predicting a 10-year risk of CVD occurrence in Iranian population and can be used in an attempt to develop national guidelines for the CVD management.

## Introduction

Recent guidelines on the primary prevention of cardiovascular disease (CVD) in clinical practice stress the urgency of having a necessary preventive intervention procedure regarding the absolute risk of CVD rather than assessment of any particular risk factors such as blood pressure and/or cholesterol levels [1]. Indeed, researchers have started to believe that hypertension is not a proper term to use. Also, the term “risk” should be applied instead of “risk factors” by the future Clinicians [2, 3]. However, most cardiovascular risk factors cannot be categorized based on the presence or absence of the risk, mostly synergistic effects of the risk factors should be considered; there is no absolute risk and the effects of the risk factors are proportional. Data from a North American study evaluating Clinicians’ ability to quantify CVD risk and treatment benefits, recommends that both general practitioners and specialist physicians substantially overestimate the CVD risk and benefits of treatment [4]. As a result, the statistical prediction of future cardiovascular events has received increased attention in recent years.

Several well-known models and charts of CVD risk assessment have been developed and updated in the past five decades, including the Framingham risk score [5], the pooled cohort equations recommended by the American College of Cardiology (ACC) in 2013, American Heart Association (AHA) cardiovascular risk-assessment guidelines [1], and the SCORE [6], ASSIGN [7], Q-Risk [8], PROCAM [9] and Globorisk [10, 11] risk prediction models. A growing body of evidence indicates that risk prediction scores lead to improvement in risk management [2]. However, the constitution of risk chart is based on the risk factors outline, which is distinct in different populations. Consequently, the risk assessment charts are specific for each population and cannot be used in different populations [12]. Hence, the design of specific risk-assessment models for different populations depends on parameters measured from the local population and this seems necessary for optimizing the risk assessment for individuals within the specific population [12].

On the report of the World Health Organization, the Middle Eastern countries are anticipated to have the highest incidence of diabetes, and in case of CVD till 2020 [13, 14]. As one of the Middle Eastern countries, Iran has alarming incidence rates of CVD and the associated risk factors [15, 16]. However, there are small data from longitudinal studies, assessing the impact of CVD risk factors among Iranian population, limited to specific areas [17]. To the best of our knowledge, no CVD risk prediction score has been specifically developed for the Iranian population to date, which is vital for developing national CVD management and prevention programs. Given the importance of developing local risk assessment tools, we used the Isfahan cohort study (ICS) dataset [14], a 10-year population-based longitudinal study started in 2001 with the main goal of developing the Persian Atherosclerotic cardiovascular disease Risk Stratification (PARS) charts in Iran. The study was performed by Isfahan Cardiovascular Research Center (ICRC), a WHO-collaborating center (http://apps.who.int/whocc/Detail.aspx?cc_ref=IRA-23&cc_code=ira).

## Materials and methods

### Study population

The ICS is a longitudinal population-based study, with 6504 adult subjects aged ≥35 years at the baseline examination, enrolled in 2001 using multistage random cluster sampling [18]. The subjects living in three Iranian central areas (Arak, Isfahan, and Najafabad), enrolled in the Isfahan Healthy Heart Program (IHHP), were recruited for ICS [14, 19]. The IHHP design was previously reported [20]. Isfahan is a city with a population of 1986542, the second most populous metropolitan area in Iran after Tehran. In 2006, the population of Arak and Najafabad, was 555975 and 282430, respectively. These areas were selected because of the socioeconomic, demographic picture and health profile similarities to the other large cities in Iran. The overall prevalence of CVD was estimated as 19.4% in Isfahan [21], which was almost similar to that of Tehran, the capital of Iran (21.8%) [22].

Participants were recruited from 2001 and followed-up for at least ten years. All subjects signed the informed consent form for the experimental procedure. Ethics approval was obtained from the Isfahan Cardiovascular Research Center Ethics Committee, a WHO collaborating center in the Eastern Mediterranean Region (EMR), and Isfahan University of Medical Sciences and conformed to the Declaration of Helsinki. For controlling selection bias, the samples were randomly selected from a healthy population. The participants were selected by multistage random cluster sampling. The study population was first stratified by their living area (urban vs rural). We then randomly selected census blocks from each county and divided them into clusters. Within each cluster, households were randomly selected for enumeration. From each household, we randomly selected one eligible individual. The inclusion criteria were as follows: being Iranian, aged ≥ 19 years, mentally competent, and not pregnant. The exclusion criteria were: having a stroke, ischemic heart attack, coronary heart disease, and heart failure.

**Fig 1:**
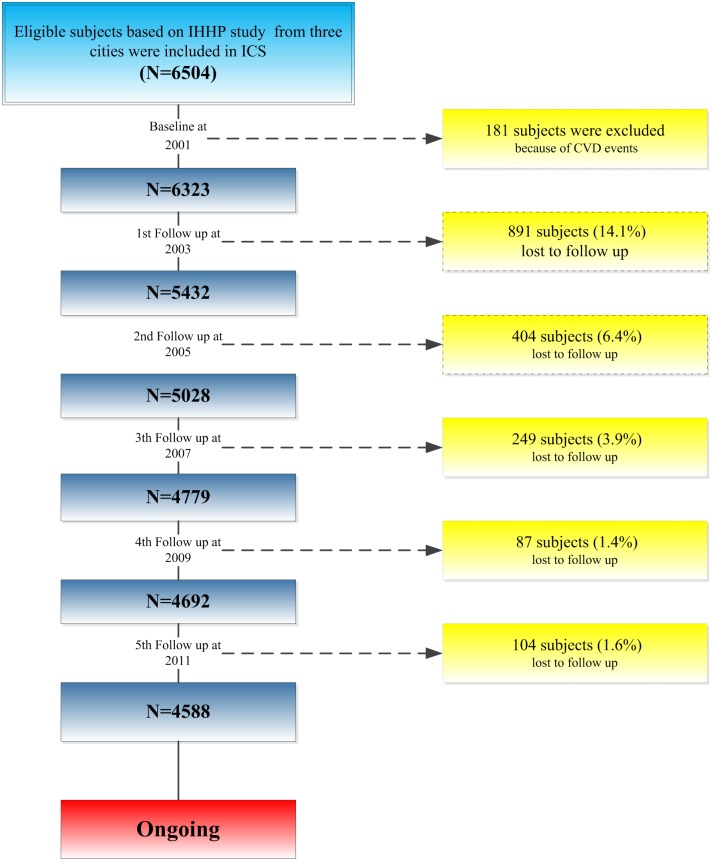
The flowchart of inclusion and lost to follow-up of ICS cohort participants.

Meanwhile, the random sampling was used without any restriction for the elderly, as to overcome underestimation or volunteer bias [16]. These subjects were followed up until CVDs occurred. All participants had no clinical history of chronic diseases and were interviewed by trained personnel (registered Nurses, Dietitians and General Practitioners), using standard questionnaires assessing lifestyle habits, various sociodemographic predictors, and clinical or biological characteristics. They were followed up by repeating these measurements every five years, and biennially by phone calls looking for the occurrence of any of primary or secondary events. However, as it happens in cohort studies, there were lost to follow-up participants in each of these follow-up phases (Fig 1). An important reason for the loss to follow-up was a change in phone numbers, based on the government new policy in the whole country. It was part of a network capacity expansion policy, without any particular distribution. It was thus completely random, not biasing the follow-up [14].

### Risk factor measurements

Participants were interviewed by trained personnel to complete standardized questionnaires, including questions on cardiovascular risk factors, and also clinical examinations, electrocardiography and laboratory evaluation [19].

After sitting for five minutes, trained physicians measured the blood pressure by standard mercury Sphygmomanometers, using the right arm of resting participants. Blood pressure measurements were repeated after 15 minutes, and the average of two measures was reported. While shoes were removed, height was measured to the closest centimeter by a trained technician. A calibrated scale was used to measure weight in light clothing. Weight (kg) was divided by height in squared meter (m^2^), and represented as body mass index (BMI). The smallest circumference at or below the costal margin was taken as waist circumference (WC). The hip circumference was taken at the level of the greater trochanter.

Fasting (12 h) blood samples (FBS) were taken from the participants. The entire samples were frozen at −20°C, in order to be assayed within 72 hours at the central laboratory of the ICRC, meeting the criteria of the WHO-collaborating center. Moreover, it was under the external quality control of St. Rafael University, Leuven and Belgium [18]. A 2-hour post-load plasma glucose (2 hpp) test was performed, using the samples from entire participants, without including diabetic subjects. DNA samples and serums were frozen at -70°C, for further analysis.

A cell counter AL820 was used to measure hematological parameters. An auto-analyzer (Eppendorf, Hamburg, Germany) was used to enzymatically measure total serum cholesterol (TC), triglycerides (TG) and FBS. In subjects with TG <400 mg/dl, the Friedewald equation was used to calculate serum low-density lipoprotein cholesterol (LDL-C), and in other cases, standard kits’ instructions were followed. Low-density and very low-density lipoproteins were precipitated, using dextran sulfate-magnesium, and then serum high-density lipoprotein-cholesterol (HDL-C) was measured [21].

### Event criteria

Since 2001, each participant has been followed up for every two years. In 2007, full structured interview, clinical and blood-sample testing were repeated as the baseline examination. Telephone interviews were implemented in 2003, 2005, 2007, 2009 and 2011 and if they were deceased, attempts were made to contact all living participants or their first-degree relatives. When the phone interviews were unsuccessful, the participants were visited at their home address for the follow-up. Structured questionnaire was asked based on being alive, hospitalized and experiencing specific neurological symptoms. The date of death, hospitalization or neurological symptoms, physician diagnosis and the hospital’s name were obtained during the interview [14]. For deaths out of hospital, death registries from the provincial mortality database were used to obtain death certificates. Verbal autopsies were performed before death by a trained Nurse based on predefined questionnaire, including medical history, signs and symptoms. Additional secondary interviews for hospitalized cases were performed where information was incomplete or inconsistent.

When there were not any inconsistent dates or diagnosis, or the records were not obtainable, we used the original medical records of MI and stroke registry database of the Surveillance Department of ICRC, to verify the reported events. If we were not able to find hospitalization data in the database, trained Nurses investigated the medical records [14].

A professional panel, consisting of Cardiologists and Neurologists have reviewed all the documents and made a decision about the diagnosis of each CVD event. Such events were defined as either acute coronary syndromes, consisting of fatal or non-fatal myocardial infarction and unstable angina, sudden cardiac death, and fatal or non-fatal stroke. The detailed description of the above-mentioned end points, risk factor measurements, and data collection have been provided in the previous reports [14, 15, 19].

### Statistical methods

#### CVD risk function

The development of the risk function in ICS was assessed, according to the following three steps: I) Refitted Framingham function: multiple Cox (proportional-hazards) regression models were derived, using the same variables of Framingham equation and ICS database. II) Recalibration of Framingham function: in these functions, the β coefficients were taken from the Framingham model, but the mean values and the incidence rates of the risk factors were taken from the ICS cohort. III) PARS function, i.e. the proposed new model in this study [5]. The regression models were first fitted one-at-a-time to a broad set of risk factors separately, including age, sex, TC, TG, systolic blood pressure (SBP), diastolic blood pressure (DBP), HDL-C, LDL-C, TC\HDL-C ratio, LDL-C\HDL-C ratio, TC\TG ratio, LDL-C\TG ratio, BMI, waist to hip ratio (WHR), WC, smoking status and diabetes. The most important risk factors were then hierarchically included in the model based on the higher hazard ratio (HR). At each step, significant risk factors remained in the model. The final model, referred to PARS risk function, had the best discrimination and calibration (The protocol: dx.doi.org/10.17504/protocols.io.j7rcrm6).

After testing the assumption of proportionality, cox proportional hazards model was used. The Schoenfeld residuals were used for testing the proportional hazards (PH) assumption for risk factors. Further evaluation was applied to check the PH assumption, regarding the risk factors plotted graphically with the log-cumulative hazard plots as a function of survival time, comparing Kaplan-Meier survival estimates and Cox adjusted estimates plotted on the same graph. If the graphical approaches suggested that there is some violation of PH assumption, an extended cox model was run based on an appropriate function of survival time. It was performed by defining the product term involving time-independent variable with some function of time (g(t) or Heaviside function) and testing the coefficient of the product term. In our analysis, there was no significant coefficient of the time-dependent variable based on product terms.

The Cox regression model was used to estimate the absolute 10-year risk of CVD (*P*) as follows:

$$\{\begin{array}{c} P=1-S{\left(t\right)}^{\text{exp}\left[f(x,M)\right]}\\ f(x,M)=\sum_{i=1}^{N}\beta_{i}\left(x_{i}-M_{i}\right) \end{array}$$ (1)

where S(t) is the survival rate calculated at the mean risk factor values, *M*_*i*_ are the mean risk factor values in the ICS, *β*_*i*_ are the regression coefficients, *x*_*i*_ represents risk factors and *N* is the number of compartments in the model.

#### Model performance assessment

A two-tailed z statistic was used to compare the hazard ratios in the ICS and Framingham functions [22, 23], in which z = (b_F_ − b_I_)/SE(b_F_ − b_I_) and $\text{SE}\left({\text{b}}_{\text{F}}-{\text{b}}_{\text{I}}\right)=\sqrt{{\text{SE}}_{{\text{b}}_{\text{F}}}^{2}+{\text{SE}}_{{\text{b}}_{\text{I}}}^{2}}$, where b_F_ and b_I_ are the β coefficient of the Framingham and ICS model, respectively with ${\text{SE}}_{{\text{b}}_{\text{F}}}$ and ${\text{SE}}_{{\text{b}}_{\text{I}}}$, as the standard errors (SEs) of b_F_ and b_I_.

The degree of discrimination power of the model was assessed, using AUROC or C-statistic and Harrell’s C. The Nam-D’Agostino chi-square test was used for evaluating time-to-event analysis calibration [24].

#### Validation procedures

Two internal validation (resampling) methods namely as 10-fold cross-validation and bootstrapping were used to obtain unbiased estimates of predictive accuracy. The above two techniques are used to assess, if the developed risk scores could be generalized to an independent data set. In this method, the data set is randomly divided into ten equal size groups. Among which, the model is tested on a single validation group, while the model is estimated, using the other nine groups (training data). Such validation is then repeated ten times, in which each of the groups is used one time, for validation. Then the average of ten validations was calculated. Moreover, internal bootstrapping was used to obtain unbiased estimates of predictive accuracy. Fifty thousand random samples were bootstrapped. Finally, overall bias in predicting CVD incidence was estimated as [13, 25]:

$$overall\overset{}{}\;bias(\%)=\left(\frac{\text{Pr}edicted\overset{}{}\;incidence-Observed\overset{}{}\;incidence}{observed\overset{}{}\;incidence}\right)\times 100$$ (2)

We applied a risk threshold, people with risk higher than 20% were considered high risk by recent guidelines [1, 26–28].

#### Risk chart

We constructed 10-year risk assessment charts of CVD incidence, using important risk factors. Such a user-friendly chart included SBP, WHR, diabetes, smoking status, CVD family history and TC. SBP was grouped into four classes: (1) <120, (2) 120–139, (3) 140–159, and (4) ≥160 mm Hg. These cutoff points were based on National Cholesterol Education Program’s Adult Treatment Panel III (ATP III). TC was categorized into five groups: (1) <150, (2) 150–200, (3) 200–250, (4) 250–300 and (5) ≥300 mg/dl. High waist-to-hip ratio (WHR) was defined as WHR ≥ 0.80 and 0.95 in women and men, respectively. When FBS ≥126 mg/dl or the 2h post-load plasma glucose ≥200 mg/dl or the patient was receiving anti-diabetic agents, the subject were diagnosed with diabetes mellitus. The smoking variable comprised of current smokers.

The following CVD probabilities (≤ 1%, 2%, 3%–4%, 5%–9%, 10%–14% and ≥15%) were displayed on the risk chart and color-coded.

Statistical modeling and analysis were performed, using SAS software, version 9.3 (SAS Institute Inc). Matlab version 8.6 (The MathWorks Inc., Natick, MA, USA) was used for risk chart generation and model validations.

Our missing data were missing at random (MAR). We thus used multivariate imputation by a chained equation’s method in STATA 12.0 for managing the measurements with missing data. The baseline characteristics and the prevalence of CVD risk factors were compared, among lost to follow-up and loyal subjects, using the sensitivity analysis. Participants without any event and loss to follow-up events were considered as censored.

## Results

The response rate of house interview was 98%, but only 95% attended the examination clinic. The main reasons for not participating were related to address and or phone number change, and to a less extent, not willing to take part in multiple follow-ups. There were 181 (2.8%) cases with CVD events excluded from the ICS baseline examination. A number of 891 (14.1%) participants were missed before the first follow-up. The loss to follow-up rate was 404 (6.4%), 249 (3.9%), 87 (1.4%) and 104 (1.6%) in the second through the fifth stage of follow-ups, respectively (Fig 1). The prevalence of the CVD risk factors and also baseline characteristics were not significantly different between lost to follow-ups and loyal subjects [14, 16].

### Baseline risk factors

The Baseline examination of participant risk factors is shown in Table 1. The Average age for men and women was 51.2±11.9 and 50.3±11.3 years, respectively. The majority of women have a high WHR (94.6%). In comparison with women, the smoking rate was far greater in men (41.6% vs. 3.3%). The SBP level has almost similar frequency distribution in men and women. Higher levels of TC were more prevalent in women, compared to men. The prevalence of diabetes mellitus was less in men, compared with women (9.3 vs. 12.6).

**Table 1:** Baseline characteristics of the study participants, ICS, 2001–2011.

	Men (N = 2648)	Women (N = 2784)
Risk factors	%	%
High waist to hip ratio^a^	39.2	94.6
Systolic blood pressure(mm/Hg)		
<120	44.4	44.9
120–139	37.1	33.7
140–159	12	13.4
>=160	6.6	8
Total cholesterol (mg/dl)		
<150	11.4	7.5
150–200	35	30.4
200–250	34.1	36.4
250–300	14.2	18.6
>300	5.2	7.2
Diabetes	9.3	12.6
Smoker	41.6	3.3
Family history of CVD	5	5.7
Age (years) (Mean±SD)	51.15±11.93	50.27±11.32

CVD: cardiovascular disease; SD: standard deviation.

^a^ Waist-to-hip ratio (WHR) ≥ 0.80 in women and ≥ 0.95 in men was considered as a high WHR.

### Cardiovascular events

A total of 705 CVD events (564 IHDs, 141 strokes) occurred, during 49452.8 follow-ups person-years (minimum 0.1, maximum 12, median 10.9 years). IHD comprised of 39 (20 women and 19 men) fatal and 113 (36 women and 77 men) non-fatal MI, 331 (171 women and 160 men) UA, and 81 (26 women and 55 men) sudden cardiac deaths. Ischemic stroke composed of 30 (16 women and 14 men) fatal and 111 cases (57 women and 54 men) of non-fatal stroke. The total CVD event rates were 1.6 per 100 person-year for men and 1.3 per 100 person-year for women, without adjusting for age. Follow-up person-years, CVD events, and the levels of risk factors of the baseline examination are shown in Table 2.

**Table 2:** Person-years of follow-up and CVD events according to risk factors in men and women, ICS, 2001–2011.

	Men		Women	
	Person-years of follow-up	CVD events	Person-years of follow-up	CVD events
Total	23931	379	25522	326
High waist to hip ratio ^a^	9190.1	214	24037.4	314
Systolic blood pressure (mm/Hg)				
<120	11132.3	96	11652	67
120–139	8741.5	148	8746.2	116
140–159	2683	76	3347.7	78
>=160	1374.1	59	1776.1	65
Total cholesterol (mg/dl)				
<150	2826.4	27	1917	15
150–200	8507.1	112	7938.5	71
200–250	8144.3	131	9206.3	123
250–300	3253.8	79	4710.25	77
>300	1198.9	30	1749.8	40
Diabetes	2059.8	82	3040.7	87
Smoker	9809.1	159	805.3	18
Family history of CVD	1205.7	22	1415.8	27

CVD: cardiovascular disease

^a^ Waist-to-hip ratio (WHR) ≥ 0.95 in men and ≥ 0.8 in women was considered as a high WHR.

### Refitted and recalibration of Framingham function

The CVD risk factor regression coefficients and HRs were estimated from sex-specific ICS regression models, using the same variables as those in the Framingham function (Table 3). During the discriminatory analysis, the AUROC was 0.730(95% CI, 0.703–0.757) and 0.754 (95% CI, 0.727–0.781), for men and women, respectively. In the calibration, the Nam-D’Agostino χ^2^ was 24.29 (p = 0.004) and 7.28 (p = 0.61) for men and women, respectively. The ICS refitted Framingham model significantly overestimated the CVD incidence with the overall bias of 149.60% and 222.70% in men and women, respectively. The 10-fold cross-validation yielded a mean AUROC of 0.73 (95% CI, 0.70–0.76) and 0.75 (95% CI, 0.73–0.78) in men and women, respectively.

**Table 3:** Comparison of relative risk and performance of ICS and Framingham.

	ICS cohort	Framingham cohort	
Women	Hazard Ratio(95% CIs)	Hazard Ratio(95% CIs)	p
Log of age	180.05(47.45–683.21)	10.27(5.65–18.64)	0.0002
Log of total cholesterol	3.26(1.02–10.38)	3.35(2.00–5.62)	0.968
Log of HDL cholesterol	0.33(0.10–1.07)	0.49(0.35–0.69)	0.516
Log of SBP if not treated	57.76(9.97–334.44)	15.82(7.86–31.87)	0.18
Log of SBP if treated	70.47(10.77–464.87)	16.82(8.46–33.46)	0.136
Smoking	1.53(0.95–2.47)	1.70(1.40–2.06)	0.697
Diabetes	1.87(1.45–2.40)	2.00(1.49–2.67)	0.734
	C statistics = 0.754 (95% CI, 0.727–0.781) χ^2^ = 7.84(p = 0.55)	C statistics = 0.793 (95% CI, 0.772–0.814) χ^2^ = 7.79 (p = 0.56)	
Men Log of age	92.37 (27.69–308.21)	21.35(14.03–32.48)	0.024
Log of total cholesterol	6.20(2.36–16.27)	3.08(2.05–4.62)	0.190
Log of HDL cholesterol	0.48(0.16–1.44)	0.39(0.30–0.52)	0.726
Log of SBP if not treated	29.08(5.58–151.54)	6.91(3.91–12.20)	0.107
Log of SBP if treated	34.32(5.72–205.92)	7.38(4.22–12.92)	0.085
Smoking	1.28(1.04–1.57)	1.92(1.65–2.24)	0.002
Diabetes	1.84(1.42–2.38)	1.78(1.43–2.20)	0.834
	C statistics = 0.730(95% CI, 0.703–0.757) χ^2^ = 20.05 (p = 0.02)	C statistics = 0.763 (95% CI, 0.746–0.780) χ^2^ = 13.48 (p = 0.14)	

The C statistic value for the recalibrated Framingham function applied to ICS was 0.700 (95% CI, 0.671–0.729) and 0.748 (95% CI, 0.721–0.775) for CVD prediction in men and women, respectively. The value of χ^2^ was 6.23 (p = 0.62) and 12.19 (p = 0.14) for men and women, respectively. The recalibrated Framingham model overestimated the CVD incidence with the overall bias of 128.23% and 176.07% in men and women, respectively. The 10-fold cross-validation, yielded a mean AUROC of 0.70 (95% CI, 0.67–0.73) and 0.75 (95% CI, 0.72–0.77) in men and women, respectively.

### PARS risk function

Having considered the variety of predictors and their interactions in a multivariate Cox regression, significant predictors of CVD events were age, sex, high WHR, SBP level, TC level, diabetes mellitus, smoking status and family history of CVD. The optimal PARS model is presented in Table 4.

**Table 4:** Adjusted HRs for CVD risk factors using PARS risk function, ICS, 2001–2011.

Risk factors	Estimate	Hazard Ratio	95% CI
Age	0.03759	1.038	1.031–1.045
Male	0.28957	1.335	1.111–1.508
Total cholesterol			
<150^a^	-	-	
150–200	0.20759	1.231	0.879–1.723
200–250	0.34201	1.408	1.013–1.957
250–300	0.45316	1.573	1.113–2.225
>300	0.54847	1.731	1.172–2.556
Systolic blood pressure			
<120^a^	-	-	
120–139	0.45643	1.578	1.291–1.929
140–159	0.73697	2.09	1.651–2.644
>=160	1.0467	2.848	2.207–3.676
Diabetes	0.63041	1.878	1.570–2.247
High waist to hip ratio^b^	0.26989	1.31	1.072–1.601
Family history of CVD	0.40182	1.495	1.116–2.002
Smoking	0.28974	1.336	1.104–1.617

CI, confidence interval;

^a^ reference category

^b^ Waist-to-hip ratio (WHR) ≥ 0.80 in women and ≥ 0.95 in men was considered as a high WHR.

**Fig 2:**
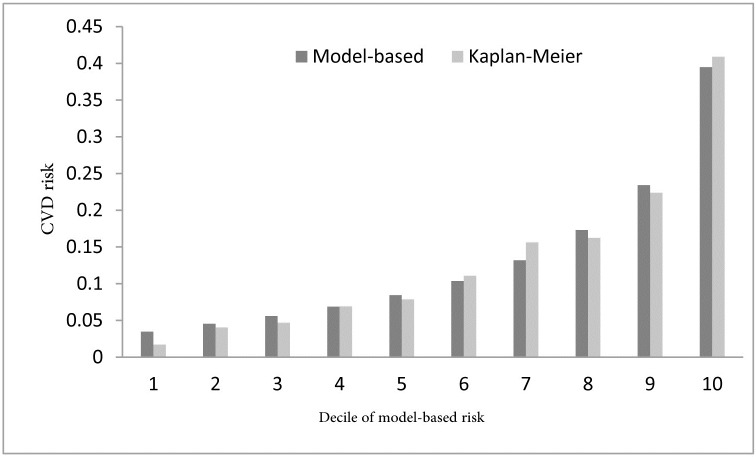
Comparison between observed and estimated 10-year risk of CVD using the PARS risk function.

The value of AUROC was 0.74 (95% confidence interval [CI], 0.72–0.76) and Harrell’s C was 0.73. In the calibration, the Nam-D’Agostino χ^2^ was 10.82 (p = 0.29). Thus, what was predicted by PARS functions was similar to the actual ICS CVD rates (Fig 2). A number of 21 evaluated risk assessment models were also shown in S1 Supporting information among which, the proposed model (Table 4) has the best goodness-of-fit in terms of the Nam-D’Agostino χ^2^ and AUROC.

**Fig 3:**
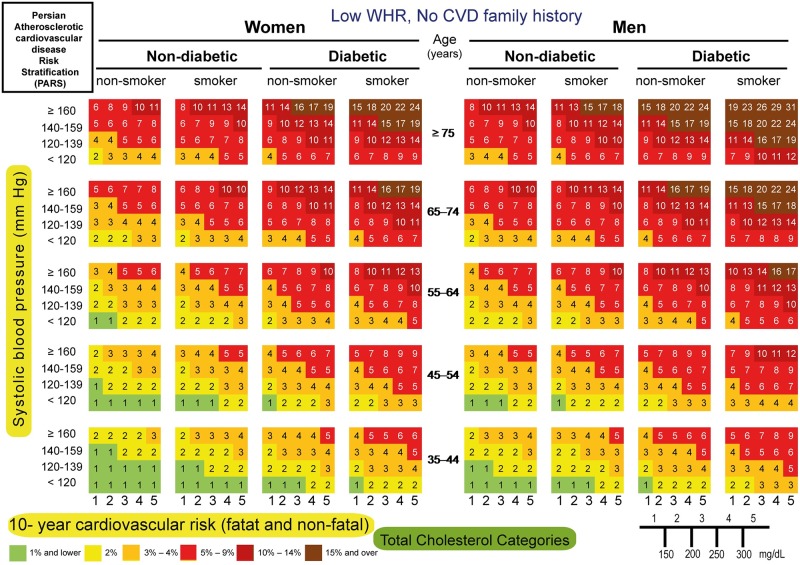
PARS charts for prediction of 10-year risk of fatal and non-fatal cardiovascular disease in ICS population with lower WHR and no CVD family history, 2001–2011.

**Fig 4:**
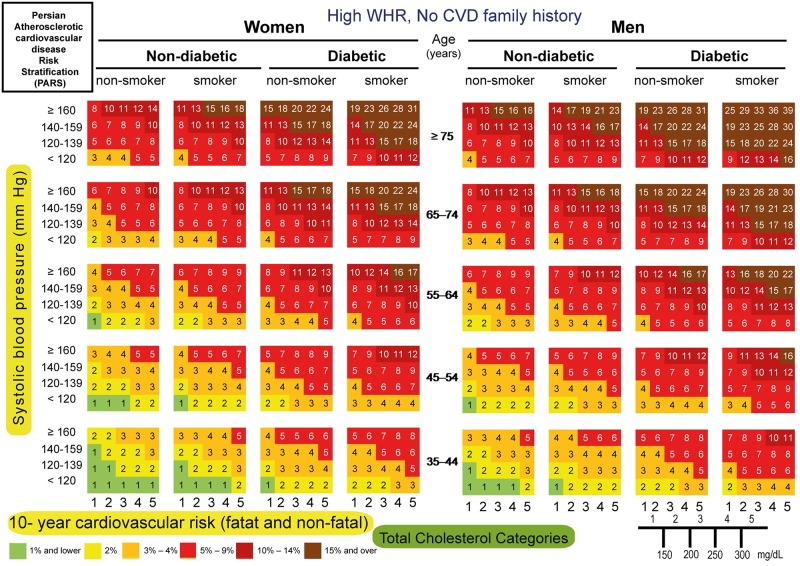
PARS charts for prediction of 10-year risk of fatal and non-fatal cardiovascular disease in ICS population with higher WHR and no CVD family history, 2001–2011.

**Fig 5:**
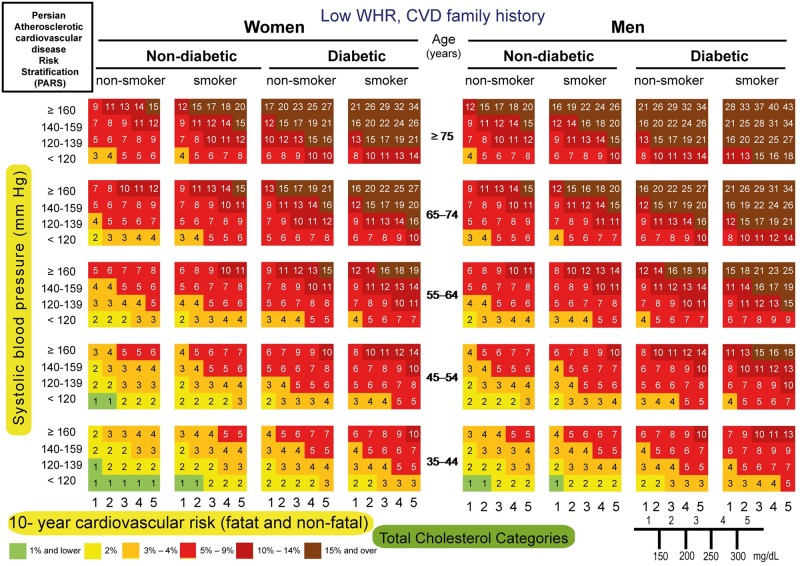
PARS charts for prediction of 10-year risk of fatal and non-fatal cardiovascular disease in ICS population with lower WHR and CVD family history, 2001–2011.

**Fig 6:**
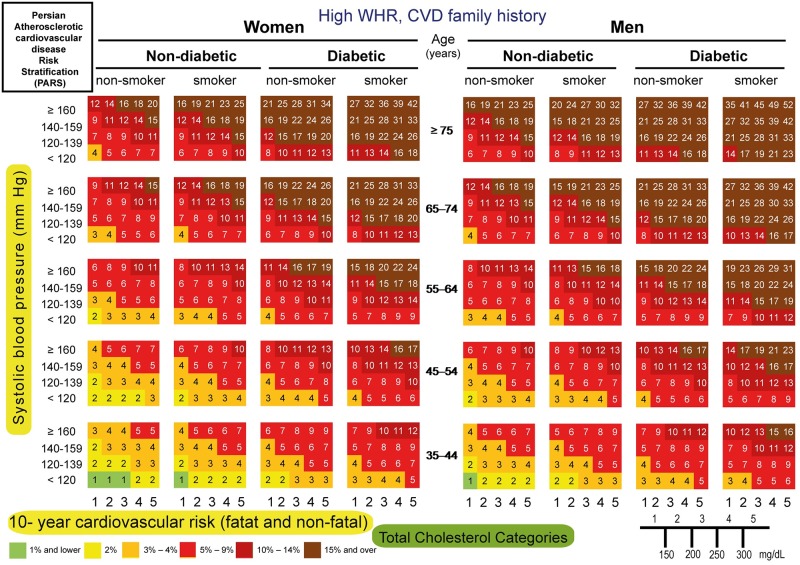
PARS charts for prediction of 10-year risk of fatal and non-fatal cardiovascular disease in ICS population with higher WHR and CVD family history, 2001–2011.

The 10-fold cross-validation, yielded a mean AUROC of 0.74 (95% CI; 0.72–0.76). In bootstrap validation, the mean AUROC was 0.74 (min-max; 0.70–0.78). Accordingly, PARS model could be internally validated. Moreover, the overall bias of the proposed model was 95.60%. PARS risk assessment charts were then created (Figs 3–6).

For instance, the estimated 10-year absolute CVD risk for a non-diabetic, non-smoker man aged 45 years, with high WHR, SBP of 145 mm Hg, TC of 205 mg/dl, and a positive family history of CVD would be 6%, as shown by the light red color (Fig 6).

## Discussion

ICS is the first longitudinal population-based study, primarily designed to develop a CVD risk assessment tool (PARS), in Iran and EMR. ICS is being organized in three geographic areas with a large urban and rural population [14]. We developed the PARS, as a new CVD risk prediction tool in Iran, in preparation for a major change in national policy, by identifying the patients at high risk of CVD.

Framingham prediction algorithms have been comprehensively used, in the United States and other countries [29–31]. However, white middle class subjects were considered in such studies. Thus, the generalized findings could not be applied, in principle, to other low- and middle-income countries [14]. Such limitations of the Framingham risk function in diverse populations were well-documented. The Framingham scores had overestimated risk in the number of populations [6, 32–34]. Consistently, our result also indicated that Framingham function overestimated risks in ICS population. We also found out that the ICS refitted and recalibrated Framingham were not so different in the prediction of CVD events. Also, central obesity and family history of CVD are independent risk factors for CVD events in the ICS population, which can be used as additive covariates, improving the model; a better calibration was found in the model.

Recently, Globorisk models, a pooled analysis of prospective cohorts and health study in different countries, in which a study from IRAN was used for validation, was introduced in the literature [10, 11]. In Globorisk models, no risk chart was provided for IRAN, and the national study used by the authors only included participants aged ≤65 years. Thus, it was not included for comparison.

Our method estimated the total CV event risk rather than the risk of CHD and/or stroke alone, not consistent with the earlier version of the Framingham risk score and the risk prediction models, originated from China [23], India [35], Turkey [36], Israel [37], Singapore [38], South Korea [39], and Chile [40]. As the aforementioned models were restricted to risk of coronary heart disease (CHD) [12] and also the PROCAM score [9], not consistent with our model, calculate the risk of CHD and stroke, separately. By calculating total CV risk, including the 10-year risk of fatal or non-fatal myocardial infarction, unstable angina, sudden cardiac death, and fatal or non-fatal stroke, we anticipate to provide a better risk estimate. Accordingly, WHO and current stress practice guidelines prefer to consider the total risk in making decisions about treatments [1, 26, 41, 42].

Based on a large Iranian representative population, we created a PARS chart. The PARS charts are user-friendly and color-coded (Figs 3–6). Furthermore, using a 6-color gradient from brown as the highest risk, to green as the lowest probability of CVD event, which help not only the Physicians but also the individuals to see their position on the chart [43].

It is known that diabetes is positively associated with the CVD risk. One of the limitations of the Framingham risk score is the small number of diabetic people in a cohort of 5573 individuals (4%) and also the diabetes definition was based on a random blood glucose concentration >9 mmol/l or the use of anti-glycemic treatment. Hence, the accuracy of the Framingham risk score for CV risk assessment in diabetic patients has been addressed in previous studies [44]. The most widely used risk score in the European countries, SCORE [6], also lacks the risk prediction power of diabetes because of the unavailability of data on diabetes, from some cohorts and also due to non-uniform definitions of diabetes, among other cohorts of the European population. In our new risk assessment charts for Iranian population, diabetes is a critical classifying risk factor due to being positioned, among the risk factors with highest HRs for the prediction of 10-year risk of CV events (Table 4). Moreover, there were 598 subjects (11%) with diabetes in our cohort (Table 1), in contrast to lower number in Framingham, making our risk estimates in diabetic patients more reliably.

While WHO [45] and the US National Heart, Lung and Blood Institute [46] recommend the assessment of WC in people with a BMI of 25·0–34·9 kg/m^2^, the majority of generally used CVD risk scores did not consider obesity measures (e.g. Framingham, SCORE, PROCAM), but it was considered only by a few scores like QRISK [47, 48]. The INTERHEART study, a large multinational retrospective study of acute myocardial infarction in 52 countries, reported that WHR was three times more strongly related to the risk of acute myocardial infarction than BMI [49]. However, these reports have not been tested in prospective studies. In our model, high WHR was accounted for significant HR (1.31), and seems to be an important risk factor in our population (Table 4). The inclusion of BMI and WC in our model or position of WHR did not improve the model performance. Our findings in this large prospective study support and expand those of the INTERHEART study, which includes data from Asian and Middle Eastern countries, in addition to western data.

It was shown in the literature that the obesity prevalence is higher in developing countries, compared with Asian countries [45]. These conclusions are mainly based on BMI values. However, if WHR is used to define obesity, such prevalence significantly increases, specifically in the Middle East [49]. Furthermore, there is strong evidence that for a given WC, Asians have higher levels of intra-abdominal adiposity, obesity-related metabolic consequences (dyslipidemia, insulin resistance, and diabetes) and mortality, compared to whites [50, 51]. It may partly justify why central obesity indices should be evaluated for CVD risk assessment in Asian and Middle Eastern countries [51]. Unlike the majority of the current risk prediction models, our data underscore the importance of WHR in the risk assessment and primary prevention of CVD (Tables 1 and 4).

We used TC because it can be measured more easily and cost-effectively than HDL or LDL cholesterol, and it is therefore measured more often in low-income and middle-income countries [10].

Our further analysis indicated other factors, including HDL-C, LDL-C, TC\HDL-C ratio, high LDL-C, LDL-C\HDL-C ratio, TC\TG ratio, LDL-C\TG ratio, and the interaction term of hypertriglyceridemia and low HDL-C, were relatively less important and were not associated with performance improvement in either gender.

Because of the following considerations, caution should be taken when the inclusion criteria of the present study are applied. (I) The study was based on the data from subjects with no CVD, and the models should not be directly applied to subjects with CVD. (II) Similar to the previous cohort studies worldwide [12], this study included individuals aged ≥35 years and therefore young adults were under-represented. Careful attention needs to be paid to the application of data for young individuals. Moreover, our charts only estimate the risk of CV event within a 10-year period.

The following ICS limitations must be considered: We underestimated the vascular diseases because stable diseases, such as heart failure, and vascular dementia and also peripheral vascular diseases were not included. Although accurate clinical data obtained from the hospital, the participants’ report of neurological symptoms was used to diagnose stroke when hospital admission was not found in the medical records. Such a verbal history is probably not accurate for CHD diagnosis [16]. Moreover, although the current study has distinctive coverage, in comparison with other similar national studies, but the samples were limited to the central area of Iran. Since, the estimated risk is dependent on the prevalence of the risk factors (Eq 1), therefore it is necessary for the proposed model to be externally validated, using other ethnic groups.

We speculate that PARS is likely to provide more appropriate estimates of CVD risk in the contemporary Iranian population with suitable discrimination for those who are at high risk in regard to age, sex, smoking status, diabetes, SBP, TC and WHR. PARS can serve as a useful tool in developing future national guidelines for the primary prevention of CVD (Figs 3–6). Furthermore, we anticipate that it could be applied in other countries in the EM region better than the western risk assessment scores.

Preparing the online prediction tool and its mobile app were also considered. The programming language used in PARS chart developed website was JavaScript and Bootstrap [52]. The JavaScript programming language has been widely used, for web programming and general purpose computing. The Android app was powered by Android Studio and API level 19 that can be used for Android 4.2 or higher version [53]. The Android app and Web-based program are freely accessible at www.prognosis.ir/PARS/index.php.

## Supporting information

S1 Supporting Information Different evaluated risk assessment models. (PDF)
