# Correction: PARS risk charts: A 10-year study of risk assessment for cardiovascular diseases in Eastern Mediterranean Region

**DOI:** 10.1371/journal.pone.0191379

**Published:** 2018-01-25

**Authors:** Nizal Sarrafzadegan, Razieh Hassannejad, Hamid Reza Marateb, Mohammad Talaei, Masoumeh Sadeghi, Hamid Reza Roohafza, Farzad Masoudkabir, Shahram Oveisgharan, Marjan Mansourian, Mohammad Reza Mohebian, Miquel Angel Mañanas

The eighth author's name is incorrect. The correct name is: Shahram Oveisgharan.

There is an error in the fourth sentence of the Abstract. The correct sentence is: Cox proportional hazard regression was used to predict the risk of ischemic CVD events defined as acute coronary syndromes, consisting of fatal or non-fatal myocardial infarction and unstable angina, sudden cardiac death, and fatal or non-fatal stroke.
