# Supplementary material for: PARS risk charts: A 10-year study of risk assessment for cardiovascular diseases in Eastern Mediterranean Region
Source: PLoS One. 2017 Dec 19;12(12):e0189389. doi: 10.1371/journal.pone.0189389 (PMC5736201; doi:10.1371/journal.pone.0189389)
Supplement: S1 Supporting Information — (PDF) [file pone.0189389.s001.pdf]

S1 Supporting Information: Different evaluated risk assessment models.

In addition to the proposed risk model (S1A table), here we also considered adding other risk factors to the original model and (or) using different risk factors (S1A-S1U tables). Models with better goodness-of-fit (i.e., less Nam-D'Agostino X^2 and higher AUROC) are preferred.

S1A: Adjusted HRs for CVD risk factors using PARS risk function, ICS, 2001-2011.

Risk factors	p-value	Hazard Ratio
Age	<.0001	1.038
Family history of CVD	0.007	1.495
Total cholestrol		
<150	-	1
150-200	0.2269	1.231
200-250	0.0417	1.408
250-300	0.0104	1.573
>300	0.0058	1.731
DM	<.0001	1.878
High WHR	0.0083	1.31
SBP		
<120	-	1
120-140	<.0001	1.578
140-160	<.0001	2.09
>160	<.0001	2.848
sex	0.0021	0.749
smoking	0.0029	1.336

Nam-D'Agostino X^2= 10.82 (p=0.29), AUROC=0.74 (0.72-0.76)

1) **PARS+ (Hypertriglyceridemia, Low HDL, High LDL, SES)**

The full model including entire lipid profiles and also Socioeconomic status (SES; see section 14 for the detailed description of its calculation) was assessed. Inclusion of Hypertriglyceridemia, Low HDL, High LDL and SES reduced the goodness-of-fit of the model (Nam-D'Agostino X^2= 14.34 (p=0.11), AUROC=0.73 (0.71-0.75)). Moreover, Hypertriglyceridemia, High LDL and SES were not significant (S1B table). Thus, we excluded High LDL (with larger p-value) at the next step.

S1B: Adjusted HRs for CVD risk factors using modified PARS risk function, ICS, 2001-2011.

Risk factors	p-value	Hazard Ratio
Age	<.0001	1.038
Family history of CVD	0.0084	1.486
Total cholestrol		
<150	-	1

150-200	0.2451	1.223
200-250	0.1094	1.366
250-300	0.0804	1.481
>300	0.0408	1.672
Hypertriglyceridemia	0.1217	1.153
Low HDL	0.0101	1.239
High LDL	0.7546	1.039
DM	<.0001	1.835
High WHR	0.0165	1.279
SBP		
<120	-	1
120-140	<.0001	1.577
140-160	<.0001	2.056
>160	<.0001	2.807
sex	0.0004	0.704
Smoking	0.0041	1.323
SES	0.5467	0.973

Nam-D'Agostino X^2= 14.34 (p=0.11), AUROC=0.73 (0.71-0.75)

2) PARS+ (Hypertriglyceridemia, Low HDL, SES)

The inclusion of Hypertriglyceridemia, Low HDL, and SES reduced the goodness-of-fit of the model (Nam-D'Agostino X^2= 14.80 (p=0.10), AUROC=0.73 (0.71-0.75)). Moreover, Hypertriglyceridemia and SES were not significant (S1C table). Thus, at next step, SES was excluded.

S1C: Adjusted HRs for CVD risk factors using modified PARS risk function, ICS, 2001-2011.

Risk factors	p-value	Hazard Ratio
Age	<.0001	1.039
Family history of CVD	0.0083	1.487
Total cholestrol		
<150	-	1
150-200	0.2354	1.228
200-250	0.0477	1.406
250-300	0.0179	1.543
>300	0.0074	1.747
Hypertriglyceridemia	0.1294	1.145
Low HDL	0.0072	1.245
DM	<.0001	1.833
High WHR	0.0165	1.279
SBP		
<120	-	1
120-140	<.0001	1.576

140-160	<.0001	2.056
>160	<.0001	2.806
sex	0.0004	0.703
Smoking	0.0041	1.323
SES	0.5484	0.973

Nam-D'Agostino X^2= 14.80 (p=0.10), AUROC=0.73 (0.71-0.75)

3) PARS+ (Hypertriglyceridemia, Low HDL)

The inclusion of Hypertriglyceridemia and Low HDL reduced the goodness-of-fit of the model (Nam-D'Agostino X^2= 15.32 (p=0.08), AUROC=0.73 (0.71-0.75)) and resulted in non-significant Hypertriglyceridemia (S1D table). Thus, at next step, Hypertriglyceridemia was excluded.

S1D: Adjusted HRs for CVD risk factors using modified PARS risk function, ICS, 2001-2011.

Risk factors	p-value	Hazard Ratio
Age	<.0001	1.040
Family history of CVD	0.0098	1.471
Total cholestrol		
<150	-	1
150-200	0.2383	1.226
200-250	0.0492	1.403
250-300	0.0182	1.541
>300	0.0080	1.737
Hypertriglyceridemia	0.1333	1.144
Low HDL	0.0073	1.244
DM	<.0001	1.839
High WHR	0.0162	1.280
SBP		
<120	-	1
120-140	<.0001	1.574
140-160	<.0001	2.051
>160	<.0001	2.807
sex	0.0004	0.711
Smoking	0.0039	1.325

Nam-D'Agostino X^2= 15.32 (p=0.08), AUROC=0.73 (0.71-0.75)

4) PARS+ (Low HDL)

The inclusion of low HDL reduced the goodness-of-fit of the model (Nam-D'Agostino X^2= 17.37 (p=0.04), AUROC=0.73 (0.71-0.75)) (S1E table). Thus, Low HDL was excluded.

S1E: Adjusted HRs for CVD risk factors using modified PARS risk function, ICS, 2001-2011.

Risk factors	p-value	Hazard Ratio
Age	<.0001	1.039
Family history of CVD	0.0084	1.481
Total cholestrol		
<150	-	1
150-200	0.1836	1.257
200-250	0.0215	1.474
250-300	0.0048	1.650
>300	0.0016	1.885
Low HDL	0.0035	1.266
DM	<.0001	1.870
High WHR	0.0118	1.294
SBP		
<120	-	1
120-140	<.0001	1.587
140-160	<.0001	2.072
>160	<.0001	2.852
sex	0.0002	0.701
smoking	0.0037	1.327

Nam-D'Agostino X^2= 17.37 (p=0.04), AUROC=0.73 (0.71-0.75)

Further evaluation was also performed considering the interaction between lipid profiles (e.g., hypertriglyceridemia and low HDL, LDL\HDL ratio, LDL\TG ratio, high Tch\HDL ratio and Tch\TG ratio).

5) PARS + interaction between hypertriglyceridemia and low HDL-C

The inclusion of interaction term of hypertriglyceridemia and low HDL-C slightly reduced the goodness-of-fit of the model, based on only X^2 criterion (Nam-D'Agostino X^2= 12.65 (p=0.18), AUROC=0.74 (0.72-0.75)) (S1F table). However, it did not show better performance than the original PARS model. Thus, we preferred a simple model without such an interaction term. This issue was also discussed in the discussion of the paper.

S1F: Adjusted HRs for CVD risk factors using modified PARS risk function, ICS, 2001-2011.

Risk factors	p-value	Hazard Ratio
Age	<.0001	1.039
Family history of CVD	0.0115	1.458
High TG. low HDL	0.0004	1.338
Total cholestrol	-	1
<150		
150-200	0.2546	1.216

200-250	0.0477	1.395
250-300	0.016	1.532
>300	0.0049	1.749
DM	<.0001	1.847
High WHR	0.0129	1.29
SBP		
<120	-	1
120-140	<.0001	1.568
140-160	<.0001	2.056
>160	<.0001	2.822
Sex	0.0004	0.714
Smoking	0.0039	1.325

Nam-D’Agostino X^2= 12.65 (p=0.18), AUROC=0.74 (0.72-0.75)

6) PARS + LDL\HDL ratio

The inclusion of LDL\HDL ratio in the model, decrease the performance of model (Nam-D’Agostino X^2= 12.38 (p=0.19), AUROC=0.73 (0.72-0.75)) and resulted in non-significant LDL\HDL ratio (S1G table). Thus, we excluded LDL\HDL ratio from the model.

S1G: Adjusted HRs for CVD risk factors using modified PARS risk function, ICS, 2001-2011.

Risk factors	p-value	Hazard Ratio
Age	<.0001	1.039
Family history of CVD	0.0072	1.493
LDL/HDL ratio	0.0604	1.078
Total cholestrol		
<150	-	1
150-200	0.4027	1.158
200-250	0.1961	1.259
250-300	0.1514	1.33
>300	0.1352	1.407
DM	<.0001	1.881
High WHR	0.0098	1.303
SBP		
<120	-	1
120-140	<.0001	1.587
140-160	<.0001	2.089
>160	<.0001	2.875
Sex	0.0034	0.759
Smoking	0.003	1.335

Nam-D’Agostino X^2= 12.38 (p=0.19), AUROC=0.73 (0.72-0.75)

7) **PARS + LDL\TG ratio**

The inclusion of LDL\TG ratio in the model, decreased the performance of the model (Nam-D'Agostino X^2= 18.24 (p=0.03), AUROC=0.73 (0.72-0.75)) and resulted in non-significant LDL\TG ratio (S1H table). Thus, we excluded LDL\TG ratio from the model.

S1H: Adjusted HRs for CVD risk factors using modified PARS risk function, ICS, 2001-2011.

Risk factors	p-value	Hazard Ratio
Age	<.0001	1.039
Family history of CVD	0.0076	1.489
LDL/TG ratio	0.3019	0.911
Total cholestrol		
<150		
150-200	0.1913	1.253
200-250	0.0298	1.447
250-300	0.0071	1.62
>300	0.0043	1.769
DM	<.0001	1.852
High WHR	0.0109	1.299
SBP		
<120		
120-140	<.0001	1.571
140-160	<.0001	2.075
>160	<.0001	2.809
Sex	0.0027	0.754
Smoking	0.0034	1.33

Nam-D'Agostino X^2= 18.24 (p=0.03), AUROC=0.73 (0.72-0.75)

8) **PARS with High Tch\HDL ratio instead of Tch**

The inclusion of High Tch\HDL ratio instead of Tch, decreased the performance of the model (Nam-D'Agostino X^2= 15.47 (p=0.08), AUROC=0.73 (0.71-0.75)) (S1I table). Thus, Tch was preferred in the model.

S1I: Adjusted HRs for CVD risk factors using modified PARS risk function, ICS, 2001-2011.

Risk factors	p-value	Hazard Ratio
Age	<.0001	1.039
Family history of CVD	0.0070	1.495
High Tch\HDL ratio	0.0006	1.300
DM	<.0001	1.899
High WHR	0.0084	1.309

SBP		
<120	-	1
120-140	<.0001	1.591
140-160	<.0001	2.092
>160	<.0001	2.902
sex	0.0073	0.777
Smoking	0.0034	1.330

Nam-D'Agostino X^2= 15.47 (p=0.08), AUROC=0.73 (0.71-0.75)

9) PARS with Tch\TG ratio instead of Tch

Inclusion of Tch\TG ratio instead of Tch in the model, decreased the performance of model (Nam-D'Agostino X^2= 20.58 (p=0.01), AUROC=0.73 (0.71-0.75)) and resulted in non-significant Tch\TG ratio (S1J table). Thus, Tch was preferred in the model.

S1J: Adjusted HRs for CVD risk factors using modified PARS risk function, ICS, 2001-2011.

Risk factors	p-value	Hazard Ratio
Age	<.0001	1.04
Family history of CVD	0.005	1.519
Tc\TG ratio	0.1871	0.912
DM	<.0001	1.915
High WHR	0.0044	1.337
SBP		
<120		
120-140	<.0001	1.586
140-160	<.0001	2.103
>160	<.0001	2.876
Sex	0.0072	0.777
Smoking	0.0033	1.331

Nam-D'Agostino X^2= 20.58 (p=0.01), AUROC=0.73 (0.71-0.75)

10) PARS with Hypertension instead of SBP

Inclusion of Hypertension instead of SBP, decreased the performance of the model (Nam-D'Agostino X^2= 16.24 (p=0.06), AUROC=0.73 (0.71-0.75)) (S1K table). Thus, we preferred SBP in the model.

S1K: Adjusted HRs for CVD risk factors using modified PARS risk function, ICS, 2001-2011.

Risk factors	p-value	Hazard Ratio
Age	<.0001	1.041
Family history of CVD	0.0123	1.452
Total cholestrol		

<150	-	1
150-200	0.1444	1.284
200-250	0.0239	1.46
250-300	0.0062	1.621
>300	0.0028	1.809
DM	<.0001	1.896
Hypertension	<.0001	1.756
High WHR	0.0027	1.358
Sex	0.0003	0.712
Smoking	0.0044	1.32

Nam-D'Agostino X^2= 16.24 (p=0.06), AUROC=0.73 (0.71-0.75)

In addition, other anthropometric measurements were assessed such as waist circumference (WC) and BMI. The resulting models are shown as below.

11) PARS with High WC instead of High WHR

The inclusion of High WC instead of High WHR, slightly decreased the performance of model (Nam-D'Agostino X^2= 10.83 (p=0.29), AUROC=0.73 (0.71-0.75)) and resulted in non-significant WC (S1L table). Thus, we preferred WHR in the model.

S1L: Adjusted HRs for CVD risk factors using modified PARS risk function, ICS, 2001-2011.

**Risk factors**	**p-value**	**Hazard Ratio**
Age	<.0001	1.039
Family history of CVD	0.0059	1.507
Total cholestrol		
<150	-	1
150-200	0.2225	1.233
200-250	0.0374	1.419
250-300	0.0086	1.592
>300	0.0047	1.755
DM	<.0001	1.903
High WC	0.2165	1.129
SBP		
<120	-	1
120-140	<.0001	1.581
140-160	<.0001	2.107
>160	<.0001	2.897
Sex	0.0168	0.805
Smoking	0.0036	1.327

Nam-D'Agostino X^2= 10.83 (p=0.29), AUROC=0.73 (0.71-0.75)

12) PARS with BMI instead of High WHR

Inclusion of BMI instead of High WHR, slightly decreased the performance of model (Nam-D'Agostino X^2= 10.96 (p=0.44), AUROC=0.73 (0.71-0.75)) (S1M table). Also, it resulted in non-significant BMI. Thus, we preferred WHR in the model, as an important risk factor in Iranian population. This issue was discussed in the discussion section of the paper.

S1M: Adjusted HRs for CVD risk factors using modified PARS risk function, ICS, 2001-2011.

Risk factors	p-value	Hazard Ratio
Age	<.0001	1.04
BMI		
<20	-	1
20-25	0.0843	1.168
>25	0.4491	1.087
Family history of CVD	0.0064	1.501
Total cholestrol		
<150	-	1
150-200	0.2221	1.233
200-250	0.0386	1.416
250-300	0.009	1.588
>300	0.0051	1.747
DM	<.0001	1.903
SBP		
<120	-	1
120-140	<.0001	1.578
140-160	<.0001	2.106
>160	<.0001	2.904
Sex	0.0341	0.833
Smoking	0.0028	1.338

Nam-D'Agostino X^2= 10.96 (p=0.44), AUROC=0.73 (0.71-0.75)

We further assessed the continuous form of risk factors. Such models had less goodness-of-fit in terms of Nam-D'Agostino X^2 and AUROC, compared with the original PARS model. Such models are mentioned as the following.

13) Extra PARS model (continuous risk factors)

S1N: Adjusted HRs for CVD risk factors using modified PARS risk function, ICS, 2001-2011.

Risk factors	p-value	Hazard Ratio
Age	<.0001	1.04
DM	<.0001	1.915
Tch	<.0001	1.003
HDL	0.0182	0.991
SBP	<.0001	1.015
Sex	0.0806	0.861
Smoking	0.0057	1.308

Nam-D'Agostino X^2=15.90, AUROC =0.73 (0.71-0.75)

S1O: Adjusted HRs for CVD risk factors using modified PARS risk function, ICS, 2001-2011.

Risk factors	p-value	Hazard Ratio
Age	<.0001	1.038
DM	<.0001	1.854
Tch	<.0001	1.003
WHR	0.0057	4.439
HDL	0.029	0.992
SBP	<.0001	1.014
Sex	0.0676	0.855
Smoking	0.0053	1.31

Nam-D'Agostino X^2=23.17, AUROC =0.73 73 (0.72-0.75)

S1P: Adjusted HRs for CVD risk factors using modified PARS risk function, ICS, 2001-2011.

Risk factors	p-value	Hazard Ratio
Age	<.0001	1.04
Family history of CVD	0.0062	1.502
DM	<.0001	1.908
Tch	<.0001	1.003
HDL	0.0196	0.991
SBP	<.0001	1.015
Sex	0.0758	0.859
Smoking	0.0062	1.304

Nam-D'Agostino X^2=19.32, AUROC =0.73 (0.72-0.75)

S1Q: Adjusted HRs for CVD risk factors using modified PARS risk function, ICS, 2001-2011.

Risk factors	p-value	Hazard Ratio
Age	<.0001	1.039

Family history of CVD	0.0073	1.49
DM	<.0001	1.85
Tch	<.0001	1.003
WHR	0.0065	4.349
HDL	0.0312	0.992
SBP	<.0001	1.014
Sex	0.0629	0.853
Smoking	0.0058	1.307

Nam-D'Agostino X^2=22.27, AUROC =0.73 (0.72-0.76)

S1R: Adjusted HRs for CVD risk factors using modified PARS risk function, ICS, 2001-2011.

Risk factors	p-value	Hazard Ratio
Age	<.0001	1.039
DM	<.0001	1.936
Tch	<.0001	1.003
SBP	<.0001	1.015
Sex	0.0431	0.842
Smoking	0.0044	1.318

Nam-D'Agostino X^2=14.34, AUROC =0.73 (0.71-0.75)

S1S: Adjusted HRs for CVD risk factors using modified PARS risk function, ICS, 2001-2011.

Risk factors	p-value	Hazard Ratio
Age	<.0001	1.038
DM	<.0001	1.869
Tch	0.0002	1.003
WHR	0.0036	4.783
SBP	<.0001	1.014
Sex	0.036	0.837
Smoking	0.0041	1.32

Nam-D'Agostino X^2=19.40, AUROC =0.73 (0.72-0.75)

S1T: Adjusted HRs for CVD risk factors using modified PARS risk function, ICS, 2001-2011.

Risk factors	p-value	Hazard Ratio
Age	<.0001	1.04
Family history of CVD	0.0057	1.508
DM	<.0001	1.929
Tch	<.0001	1.003
SBP	<.0001	1.015

Sex	0.0403	0.84
Smoking	0.0049	1.314

Nam-D'Agostino X^2= 16.54, AUROC =0.73 (0.72-0.75)

S1U: Adjusted HRs for CVD risk factors using modified PARS risk function, ICS, 2001-2011.

Risk factors	p-value	Hazard Ratio
Age	<.0001	1.038
Family history of CVD	0.0067	1.497
DM	<.0001	1.865
Tch	0.0003	1.003
WHR	0.0042	4.693
SBP	<.0001	1.014
Sex	0.0334	0.835
Smoking	0.0046	1.316

Nam-D'Agostino X^2= 22.94, AUROC = 0.74 (0.72-0.76)

14) The calculation of SES in our model

We quantified socioeconomic status (SES) based on factor analysis. We used factor analysis with only one extracted factor to obtain factor score. The original variables used as inputs of factor analysis including occupation type, education level and income, were determined based on a combination of NS-SEC model (National Statistics Socio-Economic Classification), other similar studies, and WHO recommendation on measuring socioeconomic inequalities in health [1,2]. The number of people aged under 18 and over 65 and the number of those aged 18 to 65 at home were also added as to consider the population that is economically dependent on the active age group. The occupation type was classified using the following categories: "upper white-collar employees", "lower white-collar employees", "manual workers", "self-employed persons", "unemployed", "retired", and "housewife" groups. We categorized education level as illiterate, primary school, guidance school, high school, associate or bachelor degree, and master's degree or higher. Income was categorized into five following groups, <5 million RIALs (Iranian currency unit), 5-9 million RIALs, 9-15 million RIALs, 15-20 million RIALs and >20 million RIALs [3].

References:

1. Barbeau EM, Krieger N, Soobader M-J. Working class matters: socioeconomic disadvantage, race/ethnicity, gender, and smoking in NHIS 2000. Am J Public Health 2004;94:269–78.
2. Kunst A, Mackenbach JP, World Health Organization. Regional Office for Europe. Measuring socioeconomic inequalities in health. Copenhagen: World Health Organization Regional Office for Europe; 1995.
3. Hassannejad R, Kazemi I, Sadeghi M, Mohammadifard N, Roohafza H, Sarrafzadegan N, et al. Longitudinal association of metabolic syndrome and dietary patterns: A 13-year prospective population-based cohort study. Nutr Metab Cardiovasc Dis n.d. doi:https://doi.org/10.1016/j.numecd.2017.10.025.
